# Gait and cognitive function in the elderly with AD dementia, MCI and healthy

**DOI:** 10.1002/alz.083970

**Published:** 2025-01-03

**Authors:** Seok Woo Moon

**Affiliations:** ^1^ Konkuk University School of Medicine, Chungju, Chungbuk‐do Korea, Republic of (South)

## Abstract

**Background:**

The purpose of this study was to compare gait pattern and cognitive function among elderly patients with Alzheimer’s Disease (AD), elderly people with Mild Cognitive Impairment (MCI), and Healthy Controls (HC).

**Method:**

Twenty three elderly patients participated: 25 AD (78.4±6.37 yrs), 25 MCI (74.9±6.31 yrs), and 30 HC (73.8±5.45 yrs). Gait and Cognitive function were collected using an accelerometer attached to the foot and the Korean version of the Consortium to Establish a Registry for Alzheimer’s Disease (CREAD‐K), respectively. To compare differences in gait performance among groups, mean stride time, magnitude and structure of gait variability and ratio of low frequency and high frequency (LF/HF ratio) of stride time sequence were used in this study.

**Result:**

Results showed that gait variables (mean stride time, LF/HF ratio of stride time) were useful for classification between MCI and HC. Cognitive function tests (J1, J3, J4, J6, and J8 of CREAD‐K) represented the difference between AD and HC.

**Conclusion:**

This study may provide a foundation for future work on progression of dementia.

